# Dactolisib (NVP-BEZ235) toxicity in murine brain tumour models

**DOI:** 10.1186/s12885-016-2712-4

**Published:** 2016-08-19

**Authors:** I. A. Netland, H. E. Førde, L. Sleire, L. Leiss, M. A. Rahman, B. S. Skeie, C. H. Gjerde, P. Ø. Enger, D. Goplen

**Affiliations:** 1 Oncomatrix research lab, Department of Biomedicine, University of Bergen, Jonas Lies vei 91, 5009 Bergen, Norway; 2 Neuro Clinic, Haukeland University Hospital, Jonas Lies vei 71, 5053 Bergen, Norway; 3 Department of Clinical Medicine, K1, University of Bergen, Jonas Lies vei 87, 5021 Bergen, Norway; 4 Department of Neurosurgery, Haukeland University Hospital, Jonas Lies vei 1, 5021 Bergen, Norway; 5 Kristian Gerhard Jebsen Brain Tumour Research Center, Department of Biomedicine, University of Bergen, Jonas Lies vei 91, 5009 Bergen, Norway; 6 Department of Oncology, Haukeland University Hospital, Jonas Lies vei 65, 5021 Bergen, Norway

**Keywords:** Glioblastoma, Brain tumour, PI3K, Proliferation, Dactolisib, BEZ235, Patient-derived xenograft

## Abstract

**Background:**

Glioblastomas (GBMs) are highly malignant brain tumours with a poor prognosis, and current cytotoxic regimens provide only a limited survival benefit. The PI3K/Akt/mTOR pathway has been an attractive target for therapy due to its high activation in GBMs as well as other cancers. The dual pan-PI3K/mTOR kinase inhibitor dactolisib (NVP-BEZ235) is an anti-neoplastic compound currently under investigation. However, little is known about its efficacy in human GBMs. We aimed at evaluating the efficacy of dactolisib in human glioblastoma cells, as well as in murine models carrying human GBM xenografts.

**Methods:**

To assess the effect of dactolisib in vitro, MTS assay, manual cell count, BrdU incorporation and Annexin V staining experiments were used to observe growth and apoptosis. Furthermore, Akt phosphorylation (S473), a downstream target of PI3K, was explored by western blotting. Animal studies utilizing orthotopic xenograft models of glioblastoma were performed in nude rats and NOD/SCID mice to monitor survival benefit or inhibition of tumor growth.

**Results:**

We found that dactolisib in vitro shows excellent dose dependent anti-growth properties and increase in apoptosis. Moreover, dose dependent inhibition of Akt phosphorylation (S473), a downstream effect of PI3K, was observed by western blotting. However, in two independent animal studies utilizing nude rats and NOD/SCID mice in orthotopic xenograft models of glioblastoma, we observed no survival benefit or inhibition of tumour growth. Severe side effects were observed, such as elevated levels of blood glucose and the liver enzyme alanine transaminase (ALT), in addition to diarrhoea, hair loss (alopecia), skin rash and accumulation of saliva in the oral cavity.

**Conclusion:**

Taken together, our results suggest that despite the anti-neoplastic efficacy of dactolisib in glioma treatment in vitro*,* its utility in vivo is questionable due to toxicity.

## Background

Gliblastoma (GBM) is a highly infiltrative and aggressive brain tumour for which no curative treatment exists. Notably, median survival is approximately 14.6 months post diagnosis, even when patients undergo multimodal treatment combining surgery, radio- and chemotherapy [1]. Thus, the dismal prognosis for GBM patients urgently calls for new therapeutic strategies. In recent years, advances in delineating the molecular mechanisms regulating tumour biology have laid the foundation for the development of targeted drugs [2]. Typically, these compounds are directed against the signalling pathways promoting proliferation and survival [3–5]. A major challenge however, is that the activated signalling pathways in tumour cells show a considerable overlap with those of healthy somatic cells [6]. Therefore, these drugs may cause side effects and toxicity. For the same reason, new agents need to undergo careful validation, regarding both their anti-tumour properties, as well as their toxicities.

The PI3K/Akt/mTOR pathway is frequently deregulated in GBM [7], and therefore represents an attractive target for molecular therapies. Unfortunately, clinical trials of tyrosine kinase inhibitors (TKIs) in glioblastoma have, despite good tolerability, generally showed limited efficacy [8]. This may be due to several factors, such as genetic instability and escape mechanisms, as well as inefficient drug delivery and effects, pharmacokinetic properties and unacceptable side effects [8]. Glioblastoma is characterized by heterogeneity, with a redundancy of activated signalling pathways without a unifying single dominant oncogenic “driver” mutation [2]. Therefore, aiming at a single target in GBM is unlikely to succeed [9]. As such, dual inhibitors targeting several pathways may be an attractive alternative. On the other hand, combination therapy with several inhibitors is associated with increased risk of dose limiting toxicity due to drug interactions and risk of accumulated toxicity [10].

Due to structural similarities between the ATP-binding domain of the p110 subunit of PI3K and the catalytic domain of mTOR, a class of dual inhibitors of pan-PI3K and mTOR has emerged [11]. The dual pan-PI3K/mTOR kinase inhibitor dactolisib, also known as NVP-BEZ235, is a new drug within this class with potential anti-neoplastic efficacy. Currently it is under investigation for several cancers [12].

The aim of the present study was to validate dactolisib as a glioblastoma therapy in vitro and in vivo, utilizing glioma cells and clinically relevant animal models of nude rats and NOD/ SCID mice carrying intracranial tumour material of in vivo propagated human glioblastoma biopsies.

## Methods

### Cell culture

The U87 (American Type Culture Collection, Rockville, MD, USA, ATCC HTB-14) human glioblastoma cell line was maintained in DMEM medium supplemented with 10 % fetal bovine serum, 3.2 % non-essential amino acids, 100 units/ml Penicillin/Streptomycin, 400 mol/l L-glutamine (all Sigma-Aldrich, St.Lous, MO, USA) and 0.005 mg/ml Plasmocin (InvivoGen, San Diego, CA, USA), at 37 °C and 5 % CO_2_.

Cells from serially passaged xenograft spheroids (P3) were maintained as a monolayer in NB medium (Thermo Fisher Scientific Corporation, Carlsbad, CA, USA) with the addition of 32 IE/ml heparin, 20 ng/ml bFGF and 20 ng/ml EGF (Millipore Corporation, Billerica, MA, USA).

For in vitro assessment of dactolisib efficacy, a 10 mM stock solution was prepared by dissolving dactolisib (kindly provided by Novartis (Basel, Switzerland): Also, dactolisib was obtained from Selleckchem (Houston, TX, USA) in 100 % DMSO (Sigma Aldrich, St. Louis, MO, USA). Further dilution was done in cell culture medium.

### Patient tumour material

In our study, we used a GBM xenograft model (P3) previously described [13]. This model reflects the growth pattern of human tumours in situ, including extensive infiltration into the brain parenchyma, prominent angiogenesis, and necrosis. In short, tumour biopsy tissue was obtained from the operating theatre, Haukeland University Hospital, Bergen, after approval from the regional Ethical Board and consent from patient. Tumour material was then cut into smaller pieces and maintained in medium to make spheroids [14], which again were serially passaged in rodents as described by Wang and colleagues [13]. In our experiment, the spheroids were enzymatically dissociated at 37 °C by trypsin-EDTA (Sigma-Aldrich, St. Louis, MO, USA) and DNase (Roche, Basel, Switzerland) for implantation in rats. The cells were resuspended in sterile PBS with 25 mM glucose (both Sigma Aldrich, St. Louis, MO, USA) and kept on ice until implantation of 100 000 cells in each animal. Three spheroids ranging in size between 500 and 600 μm in diameter were used for implantation in each mouse. The spheroids were kept in sterile, ice cold PBS with 25 mM glucose until implantation.

### Cell viability (MTS assay)

1000 U87 cells or 5000 P3 xenograft cells were seeded in 96-well plates 24 h prior to dactolisib exposure at following concentrations: 0, 1, 10, 20, 30, 40, 50 and 250 nM. After 72 h, the cells were analyzed using the MTS viability assay according to the manufacturer’s protocol (CellTiter 96® AQ_ueous_ One Solution Cell Proliferation Assay, Promega, Madison, WI, USA), and absorption was measured at 490 nm using a plate reader (Asys UVM340, Biochrom, Cambridge, UK). Viability was determined relative to untreated controls. These data were used to make dose response curves for determination of IC50 values in GraphPad 6 Prism (GraphPad Software Inc., La Jolla, CA, USA).

### BrdU-pulsing

Cells exposed to 0, 10, 50, 250 and 1000 nM dactolisib for 72 h, were treated with 10 μM BrdU (Sigma-Aldrich, St. Louis, MO, USA) in medium for 45 min at 37 °C. They were detached using a cell scraper, washed once with 1xPBS and resuspended to a concentration of 1 × 10^5^ cells/ml. Cell suspensions were kept on ice and processed within minutes. One hundred microliter cell suspension from each sample was loaded into individual sample chambers and centrifuged in a Shandon CytoSpin centrifuge (Thermo Fisher Scientific, Wilmington, DE, US) at 800 rpm for 3 min. Immobilized cells were fixed (described in the ICC-section below), and subsequently subjected to immunocytochemistry, imaging and quantification. For each slide, three randomly picked areas (832 μm × 665.6 μm, 554 mm^2^) were selected for quantification. The FITC stained cells and the total number of cells was manually counted, and the proportion of FITC positive cells was calculated.

### Immunocytochemistry (ICC)

Cells on coverslips in 24-well plates were fixed in 4 % paraformaldehyde (Thermo Fisher Scientific Corporation, Carlsbad, CA, USA) for 10 min, permeabilized by 0.5 % Triton X-100 (Sigma-Aldrich, St. Louis, MO, USA) in PBS for 4 min and incubated with blocking buffer (0.5 % BSA (Sigma-Aldrich, St. Louis, MO, USA) in PBS) for 15 min. All steps were performed at room temperature. Cells were incubated with primary antibodies overnight at 4 °C, in a humid atmosphere. The primary antibodies used were total Akt, pAkt S473, pAkt T308 (All Cell Signaling Technology, Danvers, MA, USA), and BrdU (Abcam, Cambridge, UK) together with DNAse (Roche, Basel, Switzerland). Following incubation, cells were washed in PBS and incubated with secondary antibodies for 45 min at 37 °C in a humid atmosphere. The secondary antibodies used were FITC-conjugated goat anti-mouse IgG1 and FITC-conjugated goat anti-rabbit (both from Southern Biotechnologies Associates Inc., Birmingham, AL, USA). After sequential washing with PBS and deionized water, cells were mounted with Vectashield mounting medium with DAPI (Vector Laboratories, Burlingame, CA, USA). Fluorescent images were obtained with a Nikon TE2000-E microscope (Nikon Corporation, Tokyo, Japan).

### Cell number quantitation

Cells were seeded in 96-well plates 24 h prior to exposure to dactolisib for 72 h at the following concentrations: 0, 10, 50 and 250 nM. The cells were detached enzymatically by Trypsin-EDTA solution, transferred to a Burker chamber haemocytometer and manually counted using a light microscope.

### Immunoblotting (Western blot)

Cell lysates were prepared by resuspending mechanically harvested cells or finely minced tissue in kinexus buffer (20 mM MOPS, 5 mM EDTA, 2 mM EGTA, protease- and phosphatase inhibitor tablets (Roche, Basel, Switzerland)), followed by Vibra-Cell sonication (Sonics & Materials Inc, Newton, CT, USA) for 3 × 5 s. Protein concentrations were determined using a Pierce BCA Protein Assay Kit (Thermo Fisher Scientific Corporation, Carlsbad, CA, USA). 20 μg lysate was mixed with NuPAGE LDS sample loading buffer and NuPAGE sample reducing agent (both Thermo Fisher Scientific Corporation, Carlsbad, CA, USA) and incubated at 70 °C for 10 min. Samples were run on a pre-cast SDS-gel (NuPage, Invitrogen, Thermo Fisher Scientific Corporation, Carlsbad, CA, USA) at 200 V for 60 min. Transfer to a nitrocellulose membrane was done at 30 V for 80 min. Following blocking in 5 % (w/w) Difco Skim milk powder (Becton, Dickinson and Company, Franklin Lakes, NJ, USA), in TBST (50 mM Tris, 150 mM NaCl, 0.05 % Tween20) for 1 h at room temperature, the membrane was incubated with primary antibody (total Akt, pAkt S473, pAkt T308 (All Cell Signaling Technology, Danvers, MA, USA) and β-actin (Santa Cruz Biotechnology Inc, Dallas, TX, USA) or GAPDH (Abcam, Cambridge, UK) at 4 °C O/N. The membrane was washed with TBST before incubation with the secondary antibodies goat anti-mouse IgG-HRP (Santa Cruz Biotechnology Inc, Dallas, TX, USA) and goat anti-Rabbit IgG (H + L) Cross Adsorbed Secondary Antibody, HRP conjugate (Thermo Fisher Scientific Corporation, Carlsbad, CA, USA) for 1.5 h. For detection, the Supersignal West Femto Maximum Sensitivity Substrate (Pierce Biotechnology, Rockford, IL, USA) was used, and chemiluminescent detection was obtained by a Fuji LAS 3000 Imager (Fuji Photo Film, Tokyo, Japan). Densitometric quantification of the bands was done using ImageJ software (National Institutes of Health, Bethesda, MA, USA).

### Annexin V / Propidium Iodide (PI) apoptosis assay

Cells were stained with the Annexin V apoptosis assay according to the manufacturer’s protocol (Thermo Fisher Scientific Corporation, Carlsbad, CA, USA). Briefly, cells were detached and washed twice by PBS (without calcium and magnesium) and once in Annexin V binding buffer. Samples were resuspended in 100 μl Annexin V binding buffer and 5 μl Annexin V Alexa Fluor 488 and 1 μl PI was added before incubation in the dark for 15 min at RT. Four hundred microliter Annexin V binding buffer was then added to each sample and the samples were kept on ice and analysed immediately on the Accuri C6 (BD Biosciences) flow cytometer.

### Animals

The in vivo studies were performed on a total number of 33 athymic homozygous nude rats (Han: nru/nru Rowett) and 32 NOD/SCID mice (NOD.CB17-Prkdc^Scid^). Animals were bred and maintained in animal facility at University of Bergen, certified by AAALAC international. The animals were provided a standard pellet diet and tap water ad libitum. They were kept in a pathogen free environment at a constant temperature and humidity and standard 12/12 h light and dark cycle.

Prior to tumour implantation, all animals were anaesthetized with isoflurane gas (Abbott Laboratories, Abbot Park, IL, USA) (3 % mixed with 50 % air and 50 % O_2_) and given Marcaine (AstraZeneca, London, England) subcutaneously. The head was secured in a stereotactic frame (Benchmark, Neurolab, St Louis, MO, USA) before a longitudinal incision was made in the scalp. Through a burr-hole obtained with a micro-drill, the tumour material was slowly inserted via a Hamilton syringe with an inner diameter of 810 μm, at the following coordinates for rats: 1 mm posterior of the bregma suture, 2 mm right of the sagittal suture and 3 mm below the brain surface. For mice, the coordinates were 0.5 mm posterior of the bregma suture, 1.5 mm right of the sagittal suture and 1.5 mm below the brain surface. The skin incision was closed using an Ethilon 3-0 suture. Animals were weighed five times a week on a PGW 2502e weight (Adam Equipment, Danbury CT, USA), inspected daily and euthanized by CO_2_ inhalation at the onset of symptoms such as passiveness, neurological deficits or other signs of illness. The brains were harvested, snap frozen in liquid nitrogen and stored at −80 °C. All procedures and experiments involving animals in this study were approved by The Norwegian Animal Research Authority (Bergen, Norway) and is in accordance with Guide for the Care and Use of Laboratory Animals (Institute for Laboratory Animal Research, National Research Council. Washington, DC: National Academy Press, 1996).

### Animal medication

Tumour bearing animals were randomly assigned to two different groups: 1) untreated controls and 2) dactolisib treatment. The medication started by the time tumour engraftment was confirmed by MRI. Dactolisib was administered by oral gavage, using malleable oral dosing needles with silicone tips (Scanbur, Karlslunde, Denmark). Dactolisib was delivered as a suspension in 0.5 % methyl cellulose and 0.5 % Tween20 (both Sigma Aldrich, St. Louis, MO, USA), once daily, 5 days a week. Vehicle (0.5 % methyl cellulose and 0.5 % Tween 20) was equally given per os(p.o.) to the animals in the control group. Both groups received 10 ml/kg solution each treatment day.

After longitudinal observation of healthy, non-implanted animals, the dose was set to 10 mg/kg for rats. The dose 45 mg/kg for mice was determined from studies published by other groups [15, 16], but was rapidly adjusted to 25 mg/kg in the study of tumour bearing mice.

During the dactolisib-exposure of non-tumour bearing animals, dactolisib was delivered as a solution in 1 volume NMP and 9 volumes PEG300 (Both Sigma Aldrich, St. Louis, MO, USA).

### Assessment of side effects

During the daily inspection of the animals, any changes and possible side effects observed (gavage reluctance, skin rash, diarrhoea and excessive salivation) were registered. To allow for semi-quantitative comparison between the groups, the animals were scored for the presence of side effects. The total number of events for each possible side effect was summed and displayed as a histogram.

### Blood collection and analysis

Immediately post mortem, blood was collected from the aorta. The blood was transferred to an Eppendorf tube, allowed to coagulate at room temperature for 30 min and centrifuged for 10 min at room temperature, 1300 rcf. The plasma was then collected and stored at −80 °C until analysis by Sentrallaboratoriet NMBU Veterinærhøgskolen (Oslo, Norway). Blood glucose levels were measured by Accu-Chek Aviva blood glucose meter (Roche, Basel, Switzerland) by one drop of freshly collected blood.

### Magnetic resonance imaging (MRI)

The animals were anaesthetized with 3 % isoflurane, in a mixture of 50 % N_2_O and 50 % O_2_, and brain images were obtained, using a Bruker Pharmascan 7 T MR scanner (Bruker Biospin MRI GmbH, Ettlingen, Germany). For rats, a coronal T2 weighted TurboRARE sequence was acquired (TR 3500 ms and TE 36 ms), in addition to an axial T1 weighted RARE sequence (TR 1000 ms and TE 9 ms), after subcutaneous injection of contrast agent (1–2 ml of Dotarem, 279.3 mg/ml, Guerbet LLC, Bloomington IN, USA). Common for both sequences for rats was slice thickness 1 mm, FOV 3.2 cm, matrix size 256 × 256, 20 slices.

Similarly, for mice, a coronal T2 weighted TurboRARE sequence was acquired (TR 4300 ms and TE 36 ms), in addition to a coronal T1 weighted RARE sequence (TR 1000 ms and TE 9 ms), after subcutaneous injection of contrast agent (0.2 ml of Dotarem). Common for both sequences for mice was slice thickness 1 mm, FOV 2 cm, matrix size 256 × 256 and 15 slices. The tumour volumes at treatment start and on follow-up MRI were calculated in Gamma Plan (Elekta Instrument AB, Stockholm, Sweden).

### Statistical analysis

In vitro experiments were repeated three times and assessed by ANOVA with Tukey’s multiple comparion test, with a *p*-value <0.05 considered significant. Kaplan-Meier survival curves were generated in GraphPad Prism 6 (GraphPad Software Inc., La Jolla, CA, USA). Median survival times for the treatment groups were compared using the log-rank test.

## Results

### Dactolisib inhibits cell proliferation and induces apoptosis of glioblastoma cells in vitro

**Fig. 1:**
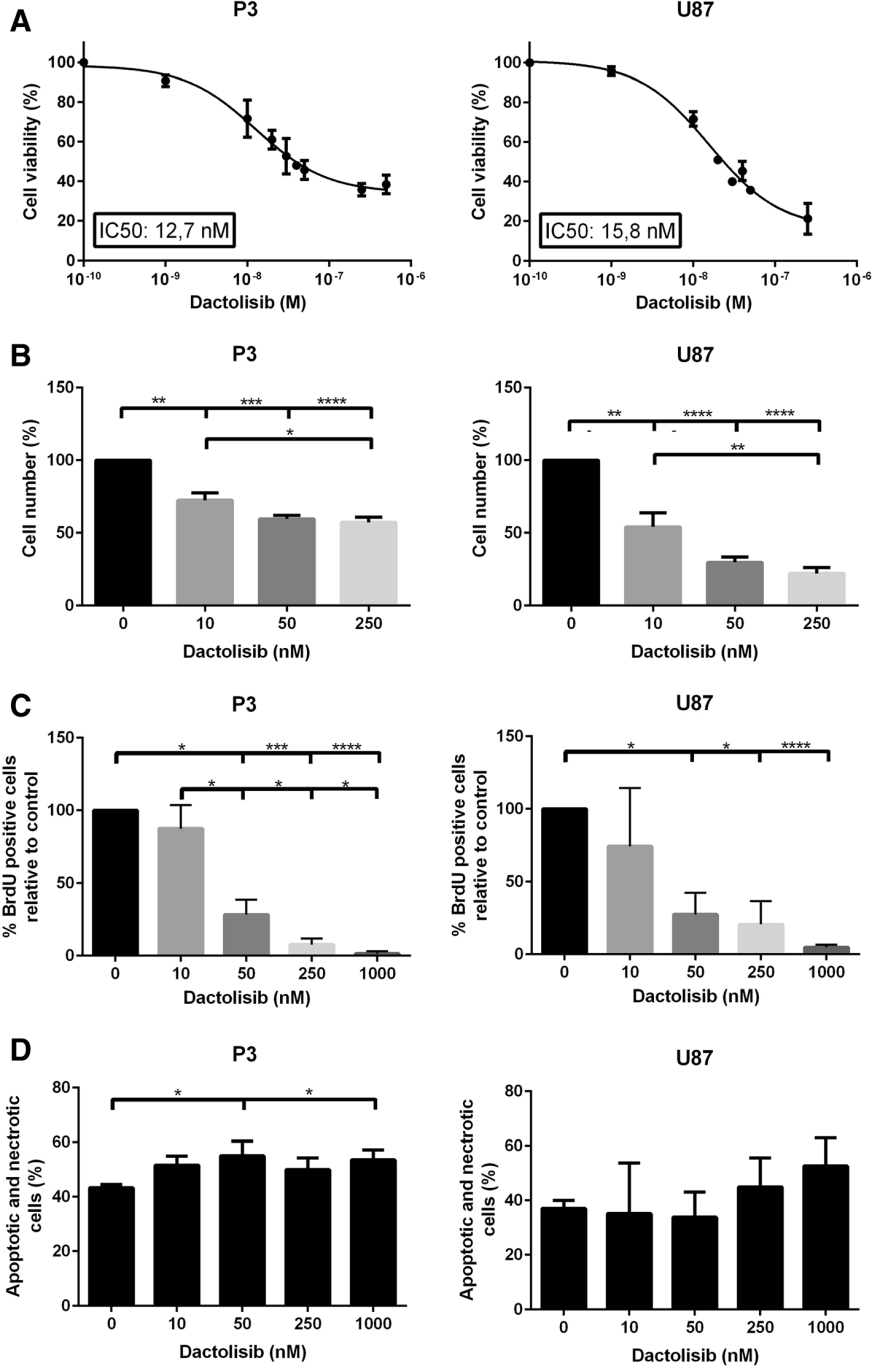
**a** IC50 doses of dactolisib for P3 (*left*) and U87 (*right*) glioma cells, generated from MTS assay. **b** Relative cell number of P3 (*left*) and U87 (*right*) glioma cells exposed to dactolisib at doses indicated for 72 h. **c** Quantification of BrdU positive P3 (*left*) and U87 (*right*) glioma cells treated with dactolisib at doses indicated for 72 h and subsequently pulsed with BrdU. **d** Quantification of Annexin V- and PI-positive P3 (*left*) and U87 (*right*) glioma cells treated with dactolisib at doses indicated for 72 h and subsequently incubated with PI and Annexin V Alexa Fluor 488 conjugate. Error bars represent s.d. of three independent experiments. *P <0.05, **P < 0.01, ***P < 0,001, ****P < 0,0001

The cytotoxicity of dactolisib on glioblastoma cells was assessed by treating the cell line U87 and monolayers established from the P3 GBM xenografts to various concentrations of dactolisib. Using the MTS viability assay, dose response curves were generated and IC50 values were calculated thereof (Fig. 1a). IC50 was established at 15.8 nM and 12.7 nM for U87 and P3 glioma cells, respectively. To rule out the possible effect of altered cell metabolism on MTS assay, we verified the cell number by manual counting (Fig. 1b). This showed that dactolisib reduced the number of cells significantly in a dose dependent manner. Since a relative reduction in cell number may reflect either increased cell death, or reduced proliferation, we quantified both the proliferation and apoptotic rates of dactolisib-treated cells. For proliferation assessment, cells were pulsed with BrdU after dactolisib exposure, and the subsequent quantification of BrdU positive cells demonstrated a clear dose dependent reduction in cell proliferation (Fig. 1c). Annexin-PI Apoptosis assay post dactolisib-exposure showed a dose-dependent increase of apoptosis (Fig. 1d). In summary, these results indicate that dactolisib is an effective inhibitor of glioblastoma cell proliferation and overall growth, as well as an inducer of apoptosis in vitro. The solvent (DMSO) did not show anti-proliferative effect on glioblastoma cells in equivalent doses alone (data not shown).

### Dactolisib inhibits phosphorylation of Akt in vitro

**Fig. 2:**
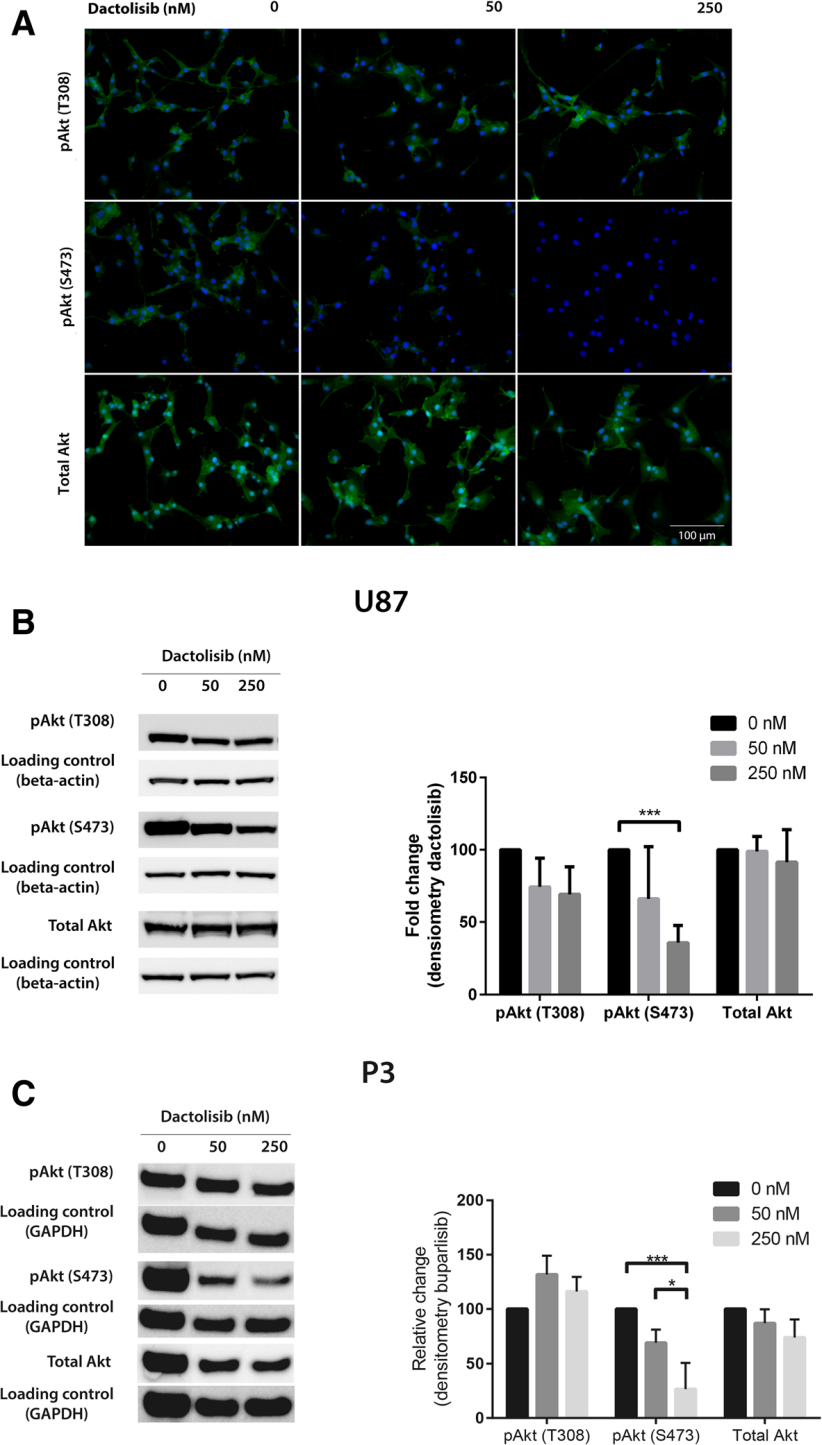
**a** Immunocytochemistry showing Akt phosphorylation in U87 cells after exposure to dactolisib at doses indicated for 72 h. *Upper panel*: Akt phosphorylated at site T308 (FITC, *green*). *Middle panel*: Akt phosphorylated at site S473 (FITC, *green*). *Lower panel*: Total Akt-levels (FITC, *green*). Nuclear counterstaining: DAPI (*blue*). **b** *Left*: Western blots showing levels of pAkt (T308), pAkt (S473) andtotal Akt in U87 cells exposed to dactolisib at doses indicated for 72 h. *Right*: Densitometric assessment of western blot, showing relative change in phosphorylation. **c** *Left*: Western blot showing levels of pAkt (T308), pAkt (S473) and total Akt in P3 cells exposed to dactolisib at doses indicated for 72 h. *Right*: Densitometric assessment of western blot, showing relative change in phosphorylation. Error bars represent s.d. of Error bars represent s.d. of three (**a** and **b**) and two (**c**) independent experiments. *P <0.05, **P < 0.01, ***P < 0,001

To further assess dactolisib’s inhibitory effect on PI3K, we evaluated the influence on Akt, a central downstream effector of PI3K. Akt is activated by phosphorylation of the amino acid residues threonine 308 (T308) and of serine 473 (S473). We performed ICC of U87 cells on coverslips exposed to various concentrations of dactolisib, suggesting a dose dependent reduction of Akt phosphorylation (Fig. 2a). For a quantitative analysis, we further performed western blot analysis of lysates from U87 (Fig. 2b) and P3 (Fig. 2c) cells exposed to dactolisib in various concentrations and assessed band intensity by densitometric analysis. A dose dependent reduction of Akt phosphorylation at S473 was observed, whereas the degree of Akt phosphorylation at T308 was unchanged. No reduction of the total levels of Akt was observed, indicating that the reduced level of phosphorylated Akt was caused by an inhibition of its phosphorylation and not by a decrease of the Akt protein level.

### Dactolisib causes adverse effects in healthy nude rats

**Fig. 3:**
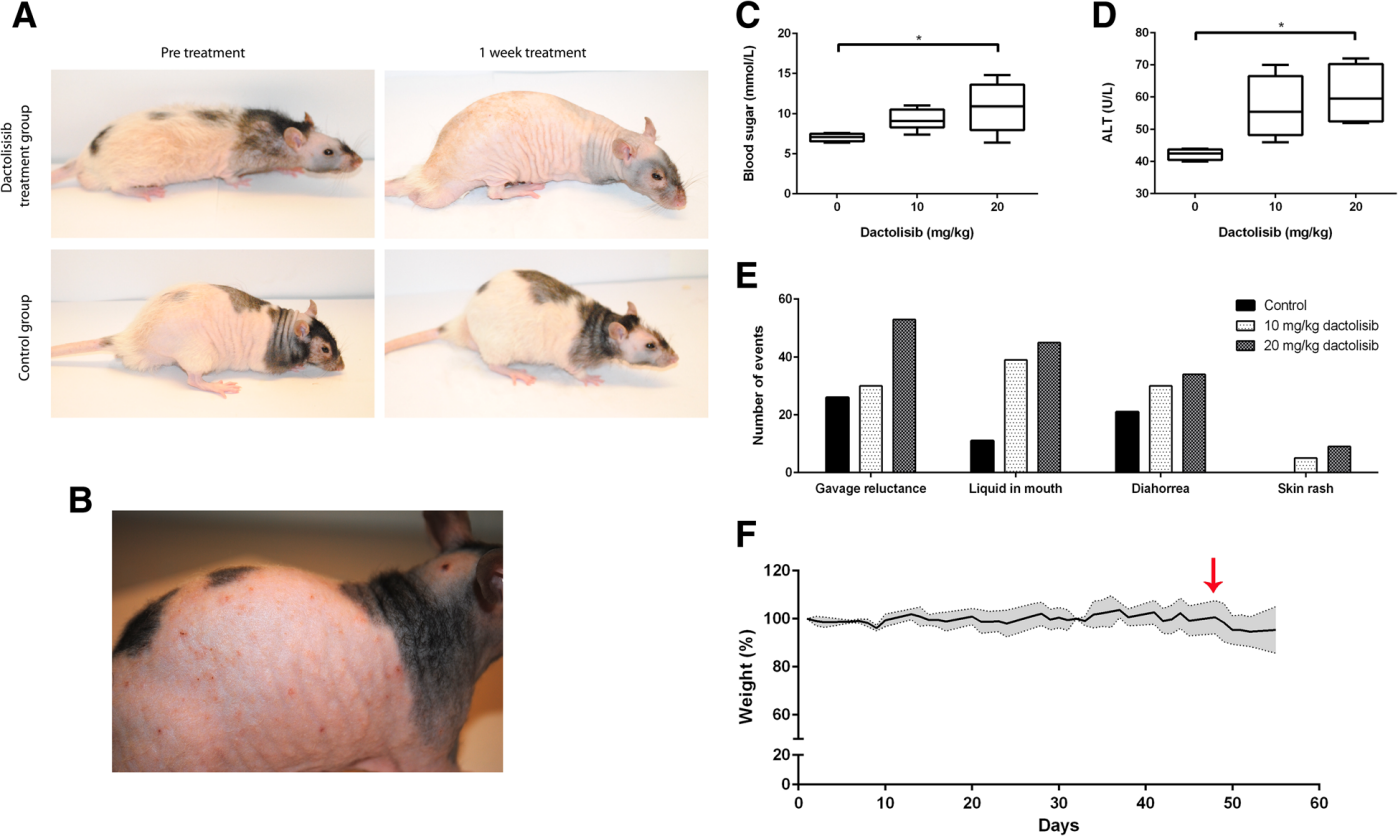
**a** Coat of nude rats before (*left panel*) and after (*right panel*) 1 week of treatment. One rat from the dactolisib treatment group (10 mg/kg) is shown in the upper panel, while one rat from the control group (vehicle only) is shown in the lower panel. **b** Maculopapular rash observed in some rats during dactolisib exposure. **c** Blood glucose levels in nude rats after 6 weeks of dactolisib treatment. *P <0.05. **d** Serum levels of ALT from rats exposed to dactolisib for 6 weeks. *P <0.05. E) Graphic presentation of adverse effects observed in nude rats exposed to dactolisib. (*n* = 6 for each group) F) Weight development in nude rats during dactolisib exposure (*n* = 4). *Red arrow* indicates dose increase from 10 mg/kg to 20 mg/kg

To assess tolerability and determine the maximum tolerated dose of dactolisib in vivo, healthy nude rats were exposed to dactolisib at doses of 10 and 20 mg/kg. After 1 week, pronounced hair loss (alopecia) was observed in all exposed rats (Fig. 3a).

The following side effects were found to appear in a dose dependent manner: Maculopapular rash (Fig. 3b and e), hyperglycemia (Fig. 3c), elevated alanine transaminase (ALT) activity in serum (Fig. 3d), and diarrhoea (Fig. 3e). Reluctance to gavage was observed in a dose dependent manner (Fig. 3e). Excess saliva in the oral cavity was observed in both treatment groups, in a dose dependent manner (Fig. 3e). Due to possible irritation of mucosae by the solvent (NMP/PEG), we also assessed the effects of the inert compound methyl cellulose as delivery vehicle. We found that the effects were less pronounced, yet still observed in a dose dependent manner when dactolisib was administered with methyl cellulose (data not shown). We thus chose to conduct the remaining studies with methyl cellulose as delivery vehicle.

Based on the unacceptable weight loss for rats under dactolisib dose escalation from 10 mg/kg to 20 mg/kg (Fig. 3f), we determined 10 mg/kg to be maximal tolerated dose (MTD) and hence this dose was applied for the study of a potential anti-proliferative effect on glioblastoma cells in vivo.

### Dactolisib does not inhibit tumour growth or prolong survival for rats carrying orthotopic GBM xenografts

**Fig. 4:**
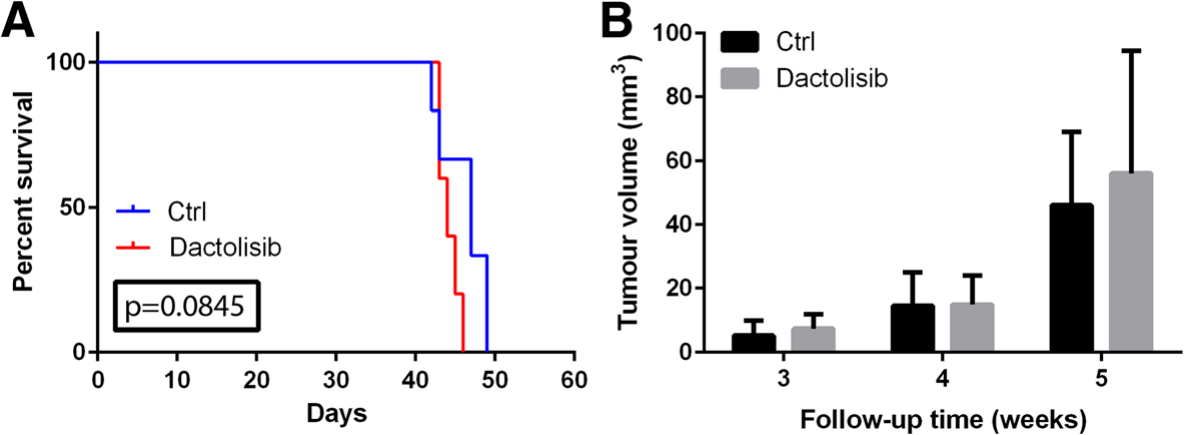
**a** Kaplan-Meyer survival curve for nude rats carrying orthotopic GBM xenografts (P3) (*p*-value 0.0845). (*n* = 6 for control group, *n* = 5 for dactolisib-group). **b** MRI-based assessment of all tumour volumes

The anti-tumour efficacy of dactolisib was evaluated in a clinically relevant patient-based GBM model. In vivo propagated P3 GBM xenografts were intracranially implanted in nude rats. This model reflects the growth pattern of human tumours in situ, including extensive infiltration into the brain parenchyma, prominent angiogenesis, and necrosis [13]. Three weeks after tumour implantation, magnetic resonance imaging (MRI) confirmed tumour engraftment in all rats, and the animals were randomly assigned to two treatment groups: one receiving dactolisib, and one receiving vehicle only (control). Dactolisib did not increase the survival of treated animals (Fig. 4a). Although not statistically significant (*p* = 0.0845), the animals in the treatment group showed a tendency towards shorter survival than the rats in the control group. MRI performed after 1 and 2 weeks of treatment showed tumours of comparable size in both groups (Fig. 4b).

### Healthy NOD/SCID mice tolerate higher doses of dactolisib than healthy nude rats

**Fig. 5:**
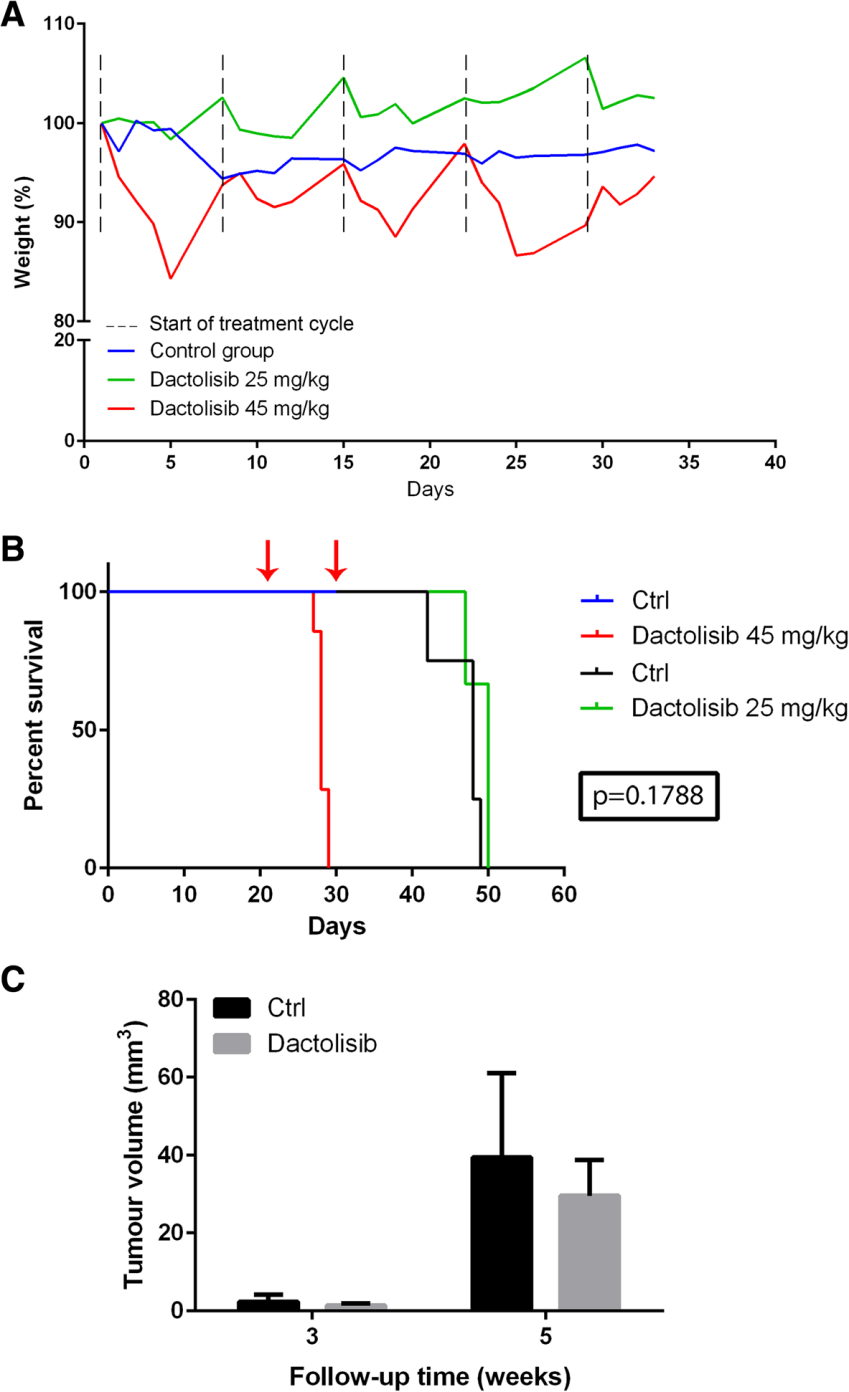
**a** Weight development in healthy, non-tumour bearing NOD/SCID mice during dactolisib exposure (*n* = 6 for each group). **b** Kaplan Meyer survival curve for NOD/SCID mice carrying orthotopic GBM xenografts (P3). *Left red* *arrow* indicates treatment start of 45 mg/kg dactolisib (*n* = 7). *Right red arrow* indicates splitting of control group (*n* = 7) into one treatment group (25 mg/kg) (*n* = 3) and one control group (*n* = 4). *Red line* shows survival for mice in the treatment group of 45 mg/kg dactolisib, *green line* shows survival for mice in the treatment group of 25 mg/kg dactolisib, and *black line* shows survival for mice in the control group (vehicle only). (*P*-value 0.1788 for 25 mg/kg vs control). **c** MRI-based assessments of all tumour volumes

The failure to show dactolisib efficacy in glioblastoma bearing nude rats, together with its excellent in vitro anti-tumour effect, suggested that 10 mg/kg dactolisib was not enough to reach a therapeutic concentration within the brain. Since our preceding experiments indicated that a higher dose was unacceptable for rats, we next investigated the effect of dactolisib on mice. Healthy, non-implanted NOD/SCID mice were administered dactolisib at doses of 45 and 25 mg/kg by oral gavage. Mice receiving 25 mg/kg dactolisib had no weight loss compared to the control group, while the group exposed to 45 mg/kg dactolisib lost weight during the treatment (Fig. 5a). However, the weight loss was within less than 15 %, and the animals normalized their weight after the 2-day rest in each treatment cycle.

### 45 mg/kg dactolisib is highly toxic for mice carrying intracranial GBM xenografts

In vivo passaged patient-derived GBM xenografts were intracranially implanted in NOD/SCID mice. Three weeks after implantation, magnetic resonance imaging (MRI) confirmed tumour engraftment in all mice, and the animals were randomly assigned to two groups: one receiving 45 mg/kg dactolisib, and one receiving vehicle only (control). However, within the first week of treatment, all mice in the treatment group died, while all animals in the control group survived (Fig. 5b).

### 25 mg/kg dactolisib does not improve survival for mice carrying intracranial GBM xenograft, and does not reduce tumour growth

After the death of all mice receiving 45 mg/kg dactolisib, half of the mice in the control group were then allocated to a new treatment group receiving 25 mg/kg dactolisib. No survival benefit was observed in the animals treated with 25 mg/kg dactolisib (Fig. 5b). MRI 1 week after initiation of the treatment with 25 mg/kg dactolisib revealed a slightly smaller, yet not statistically significant, tumour in the treatment group (Fig. 5c).

## Discussion

In the present study, we have demonstrated the in vitro efficacy of the dual PI3K/mTOR inhibitor dactolisib, with dose dependent reduction of glioblastoma cell proliferation, increased apoptosis and corresponding reduction of Akt phosphorylation.

Our in vivo studies revealed that the most prominent side effect of dactolisib treatment was hair loss (alopecia), which was regarded a sign of successful administration and absorption of the compound, since the cells within the hair follicle have a high basal level of PI3K activity [17], and its inhibition thus leads to hair loss. Moreover, dose dependent hyperglycemia was also observed. Given the well-described role of PI3K in insulin signalling [18, 19], elevated blood glucose may be an indispensable adverse effect of PI3K inhibition. Hyperglycemia is a particularly problematic side effect from a compound for glioblastoma treatment, since many GBM patients experience elevated blood glucose due to steroid treatment. Furthermore, hyperglycemia is associated with poorer survival for GBM patients [20]. An elevated serum alanine transaminase (ALT) activity was also found in the rats exposed to dactolisib. ALT serves as a biomarker of hepatotoxicity [21], and may thus indicate hepatocellular damage as a result of dactolisib exposure. Interestingly, elevated activities of serum ALT is commonly observed in diabetic patients [22]. After a few days of accustoming, rats were cooperative during gavage. However, after 3 weeks they became increasingly reluctant to drug administration. This might represent mucosal irritation, and was seen in the treatment groups as well as in the control group, yet in a dose dependent manner. Possibly, mechanical irritation by the oral dosing, together with the possible chemical irritation by the vehicle, might be the explanation for the observed mucosal irritation in the control group. Nevertheless, the dose dependent increase in mucosal irritation in the treatment group was most likely caused by dactolisib. Mucositis has also been reported as a side effect from dactolisib in a phase I clinical trial [23]. Diarrhoea was also observed across the groups, yet more often and more severe in the group of animals treated with higher the dose of dactolisib. Excess of mouth fluid, interpreted as mucositis, was observed in a dose dependent manner. Notably, all of the above described side effects have also been observed with dactolisib in clinical trials [24]. Vomiting has also been reported from these trials. However, rats and mice lack the emetic reflex [25].

For animal experiments, Novartis, the manufacturer of dactolisib, advices dactolisib administered as a suspension in methyl cellulose or as a solution in NMP/PEG. The latter is more commonly used. Hence, for study of dactolisib exposed, non-tumour bearing rats, we used NMP/PEG as vehicle in both treatment groups, as well as in the control group. However, in our experience, the viscosity of NMP/PEG results in a more tedious procedure, and we thus examined the effects of dactolisib as a suspension in methyl cellulose. Our findings indicate that NMP/PEG as vehicle for dactolisib delivery causes adverse effects or exacerbates the adverse effects of dactolisib, which are reduced if NMP/PEG is replaced by methyl cellulose.

The in vivo studies were initially performed in nude rats, as this was the animal species the GBM xenograft model we used was first established in. The failure to show efficacy of dactolisib against glioblastoma in nude rats, together with the excellent in vitro anti-tumour effect, led us to hypothesize that MTD for nude rats, 10 mg/kg dactolisib, was not high enough to reach a therapeutic concentration within the brain. We thus next investigated the effect of dactolisib on healthy, non-implanted NOD/SCID mice, and found that dosing up to 45 mg/kg was tolerable, although side effects have been found in mice treated with dactolisib for a prolonged period of time with intraperitoneal (i.p.) injections [26]. However, 45 mg/kg proved to be intolerable in mice carrying intracranial xenografts. Within the first week of treatment, all mice in the treatment group died, while all animals in the control group survived. The possible explanation is that the burden of a test substance causing adverse effects may be intolerable for the weakened animal already carrying an intracranial tumour. After the first treatment cycle, where all mice in the treatment group died, half of the animals in the control group were allocated to a new treatment group receiving lower dose of 25 mg/kg dactolisib. No survival benefit was observed with this dose and MRI 1 week after initiation of the treatment at this dose level, revealed tumours of comparable size in both groups. It is still possible that dactolisib exerts anti-tumour efficacy but that this is outweighed by its side effects. Thus, a change of the route of administration may improve the results. Since many of the side effects observed stem from the gastrointestinal tract, it might be advisable to avoid oral ingestion, or aim at reducing the irritation of the GI by carefully designed medication, such as enterocapsules. The dosage schedule is also of importance. Alcazar et al. have reported the use of dactolisib doses up to 75 mg/kg for nude mice, yet with medication every other day [27]. The tumour site may also influence the tolerance, as Klinger et al. gave 45 mg/kg/day p.o. and Yu et al. administered 25 mg/kg/day i.p. on mice with subcutaneous glioma tumours [28, 29]. Other dosage schedules have been used for other cancer types, although many use a different route of administration and/or time of exposure [30–36].

Alternatively, the lack of efficacy may also be due to limited distribution across the blood-brain-barrier (BBB). The low molecular weight of dactolisib (469.5 Da) and its lipophilic capacity satisfy the basic requirements for crossing the BBB [37], yet no documentation on crossing of BBB by dactolisib has been found to date. However, two studies report dactolisib efficacy on intracranial tumours in nude mice, indicating crossing of the BBB [16, 27]. This is in contrast to our present data, and the reason for this discrepancy is not clear. However, whereas our data were obtained in nude rats and NOD/SCID mice, nude mice were applied for these studies.

Other types of cancer may benefit from dactolisib at lower doses than those required for GBM. Of particular interest is the study of Wang et al., which reports that side effects during dactolisib-treatment of breast cancer bearing mice are reduced by the addition of dihydrotestosterone (DHT) [38]. The activation of androgen receptor (AR) by DHT is beneficial for breast cancer patients. However, AR signaling may aggravate gliomas [39], and the safety of using DHT in GBM patients is uncertain.

Currently, there are no ongoing clinical studies for the use of dactolisib in glioblastoma, yet there is one recruiting phase I/II study (NCT01508104), which includes advanced solid cancers, also covering glioblastoma. The aim of this study is to assess the safety of combination treatment with another mTOR inhibitor, everolimus.

Toxicity of therapeutic compounds in palliative cancer therapy is an important issue. To date no curative treatment option for glioma has been established. The standard therapy is associated with an acceptable side effect profile. The evaluation of clinical benefit versus toxicity is at the discretion of the oncologist. However, new therapeutic compounds should be implemented into the clinic with caution, and drugs with marginal, if any, efficacy and considerable toxicity compromising the quality of life should be avoided.

## Conclusion

Taken together, our findings of dactolisib anti-tumour efficacy in vitro support the concept of application of dual inhibitors in cancer therapy. However, the demonstrated association of a spectrum of side effects from dactolisib treatment in two different murine brain tumour models, suggest that further studies need to proceed with caution.
